# Species Differences in Stereoselective Pharmacokinetics of HSG4112, A New Anti-Obesity Agent

**DOI:** 10.3390/pharmaceutics12020127

**Published:** 2020-02-03

**Authors:** In Yong Bae, Min Sun Choi, Young Seok Ji, Sang-Ku Yoo, Kyungil Kim, Hye Hyun Yoo

**Affiliations:** 1 Institute of Pharmaceutical Science and Technology and College of Pharmacy, Hanyang University, Ansan, Gyeonggi-do 15588, Korea; iybae722@naver.com (I.Y.B.); chm2456@hanyang.ac.kr (M.S.C.); wldudtjr23@hanyang.ac.kr (Y.S.J.); 2 Glaceum Inc., Yeongtong-gu, Suwon, Gyeonggi-do 16675, Korea; skyoo@glaceum.com (S.-K.Y.); kikim@glaceum.com (K.K.)

**Keywords:** HSG4112, anti-obesity agent, stereoselectivity, pharmacokinetics

## Abstract

HSG4112, a racemic drug, is a new anti-obesity agent. In this study, the stereoselective pharmacokinetics of HSG4112 were investigated in rats and dogs, and the underlying mechanism was investigated. The plasma concentrations of HSG4112(S) and HSG4112(R) were quantitated in plasma from rats and beagle dogs after IV and/or oral administration of racemic HSG4112. The concentration of HSG4112(S) was significantly higher than that of HSG4112(R) in rat plasma. Contrarily, the concentration of HSG4112(R) was significantly higher than HSG4112(S) in dog plasma. A metabolic stability test with liver microsomes showed that HSG4112(S) was more stable than HSG4112(R) in rat liver microsomes, but the difference between stereoisomers did not appear in dog liver microsomes. However, the stereoselectivity was observed in dog liver and intestinal microsomes after uridine 5’-diphospho-glucuronic acid was added. Thus, stereoselective metabolism by uridine 5’-diphospho-glucuronosyltransferases is mainly responsible for the stereoselective pharmacokinetics in dogs. These results suggest that the species difference in the stereoselective plasma pharmacokinetics of HSG4112 is due to the stereoselective metabolism.

## 1. Introduction

**Figure 1:**
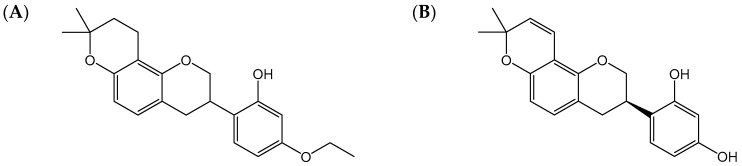
Chemical structures of (**A**) HSG4112 and (**B**) glabridin.

HSG4112 is a new drug candidate which has been developed as a treatment for obesity. Its chemical structure is derived from glabridin. Glabridin is an isoflavane, which is found in *Glycyrrhiza glabra* extract [1,2,3]. It is known to have whitening activity by suppressing the activity of tyrosinase during the synthesis of melanin and to help alleviate gastroenteric disorders. Recently, it was confirmed that glabridin is effective in metabolic syndromes, including hyperlipidemia, fatty liver, impaired glucose metabolism, diabetes, and obesity and has anti-inflammatory actions, anticancer actions, and the like [4]. However, in spite of useful medicinal efficacy, glabridin is easily broken down by sunlight, moisture, acidity, basicity, oxygen, heat, and the like due to low chemical stability, so it is very difficult to develop a product actually utilizing glabridin [4]. For these reasons, we synthesized a new pyranochromenylphenol derivative, HSG4112, by modifying the structure of glabridin. HSG4112 is stable under various physical conditions, while maintaining or improving its medicinal efficacy [4]. HSG4112 is a racemic compound with a chiral carbon. Thus, glabridin has the structure of R-enantiomer whereas HSG4112 is a mixture of S and R enantiomers (Figure 1).

In many cases with chiral drugs, one of the two enantiomers is active and the other is either non-active or even harmful [5,6,7]. This is because the interaction between a drug molecule and its target is dependent on its three-dimensional environment. The most famous example is thalidomide, which was sold in the 1950s; the drug was introduced as a racemic mixture for use as a sedative but was later withdrawn from the market following the occurrence of birth defects in the children of mothers who took it to treat morning sickness. It was later found that the inactive enantiomer was the cause of the teratogenicity. This disaster was a driving force behind the requirement to strictly test drugs before making them available to the public [8]. In particular, for racemate candidates, both enantiomers should be studied separately as early as possible to assess the relevance of stereoisomerism to effects and fate. Accordingly, a pharmacokinetic evaluation should be provided for each enantiomer [9,10].

In this study, the stereoselective pharmacokinetics of HSG4112 were investigated in rats and beagle dogs. In addition, the metabolic stability was investigated in liver and intestinal microsomes from five different species (rat, mouse, dog, monkey, and human) to evaluate the role of metabolism in the species differences in its stereoselective pharmacokinetic behavior.

## 2. Materials and Methods

### 2.1. Chemicals and Materials

HSG4112(S) and HSG4112(R) were given from Glaceum Incorporation (Suwon, Korea). Pooled rat liver microsome (RLM), mouse liver microsome (MLM), dog liver microsome (DLM), and human liver microsome (HLM) were obtained from Gentest (Woburn, MA, USA). Pooled rat intestinal microsome (RIM), mouse intestinal microsome (MIM), dog intestinal microsome (DIM), and human intestinal microsome (HIM) were obtained from Sekisui Xenotech (Kansas City, KS, USA). Glucose 6-phosphate, β-NADP+, glucose 6-phosphate dehydrogenase and alamethicin were obtained from Sigma-Aldrich (St. Louis, MO, USA). HPLC-grade acetonitrile (ACN) and formic acid were purchased from J.T. Baker (Phillipsburg, NJ, USA). Distilled water (DW) was prepared using a Milli-Q purification system (Millipore, Bedford, MA, USA).

### 2.2. Preparation of Calibration and Quality Control (QC) Standards

HSG4112(S) 10 mg, HSG4112(R) 10 mg, and HSG4112-d5 (internal standard; IS) 5 mg were weighted and transferred to a 10 mL volumetric flask and ACN was added to the marking line to completely dissolve and store in a refrigerator (4 °C). The standard stock solutions of HSG4112(S) and HSG4112(R) were serially diluted to the designated concentrations to prepare the working standard solutions. The IS stock solution was diluted to 100 ng/mL with ACN and used for plasma sample preparation. Working standard solutions (5 µL) were added to plasma (95 µL) to yield calibration standards of 5, 20, 50, 100, 500, 1000, 2000, and 5000 ng/mL. QC samples were prepared at final concentrations of 5(LOQ), 15(low), 400(mid), and 4000(high) ng/mL in the same manner as the calibration standards.

### 2.3. Animal Plasma Samples

Rat and dog plasma samples were obtained from Biotoxtech Co. Ltd. (Cheongju, Korea). Plasma samples for oral pharmacokinetic study were obtained from the 4-week repeated oral toxicity study. The information on the plasma samples were provided in the Supplementary data. Briefly, for the pharmacokinetic analysis in rats, HSG4112 was orally administered HSG4112 at a dose of 100 mg/kg/day for 4 weeks and the plasma samples were collected on the 28th day. The blood drain time point was 0, 0.5, 1, 2, 4, 6, 10, and 24 h (*n* = 3; Table S1). In addition, rats were intravenously administrated HSG4112 at a dose of 10 mg/kg and blood was collected at 0, 0.083, 0.25, 0.5, 0.75, 1, 2, 3, 6, 12, and 24 h (*n* = 3). For the pharmacokinetic analysis in dogs, HSG4112 was orally administered at a dose of 100 mg/kg/day for 4 weeks (*n* = 3), and the plasma samples were collected on the 28^th^ day. Blood samples were taken at 0, 1, 2, 4, 6, 10, 12, and 24 h. In addition, dogs were administrated HSG4112 intravenously at a dose of 2 mg/kg (*n* = 2) and blood was collected at 0, 0.083, 0.25, 0.5, 1, 2, 3, 4, 6, 12, and 24 h. All animal studies were performed according to the guidelines of the Ethics Committee for Use of Experimental Animals and approved by the Institutional Animal Care and Use Committee of Biotoxtech Co. Ltd. (Approval ID and date: 2016-05-160252, 2016-05-160276).

### 2.4. Sample Preparation

The plasma (30 μL) sample was put into a 1.5 mL tube and added with 60 μL IS solution. The tube was vortexed and centrifuged at 13,200 rpm for 5 min at room temperature. The supernatant was transferred to an LC vial for LC-MS/MS analysis.

### 2.5. In Vitro Metabolic Stability

Incubation mixture containing liver or intestinal microsomes (1 mg/mL), and HSG4112 (10 µM) in potassium phosphate buffer (0.1 M, pH 7.4) were preincubated at 37 °C for 5 min. The reactions were initiated by the addition of an NADPH-generation solution in a final incubation volume of 100 μL (*n* = 3). For glucuronide conjugate formation, incubation mixture containing microsomes (1 mg/mL), magnesium chloride (2.5 mM), UDPGA (2 mM), alamethicin (25 µg/mL), and drug (10 µM) in potassium phosphate buffer (0.1 M, pH 7.4) were preincubated at 37 °C for 5 min. The reactions were initiated by the addition of the NADPH-generation solution in a final incubation volume of 200 μL (*n* = 3). After the incubation for 0, 30, 60, and 120 min, the reaction was stopped by the addition of 200 μL ACN with IS. The samples were vortex-mixed and centrifuged at 13,200 rpm for 5 min. The supernatant (5 μL) was injected on to ultra-performance liquid chromatographic (UPLC) column for LC-MS/MS analysis. The experiment was performed in triplicate.

### 2.6. LC-MS/MS

In order to quantify HSG4112(S) and HSG4112(R), the Acquity UPLC-MS/MS system (Waters, Milford, MA, USA) was used with an electrospray ionization source. Mass detection was performed in the negative ion mode, and the column temperature was maintained at 40 °C using a thermostatically controlled column oven. The column used for the separation was a CHIRALPAK^®^ IC-U (1.6 µm, 3.0 × 100 mm; DAICEL, New York, NY, USA). The mobile phases consisted of D.W (solvent A) and ACN (solvent B). For quantification of the analytes, isocratic elution was performed at a flow rate of 0.5 mL/min. Solvent B was maintained at 60%. For multiple reaction monitoring (MRM) analyses, the target ions used were *m/z* 353.3→137.1 for HSG4112(S) and HSG4112(R) and *m/z* 358.3→142.0 for HSG4112-d5. The capillary voltage was 3 kV and cone voltage was 50 V. Collision energy was 23 V. Nitrogen was used as the desolvation gas at a flow rate of 650 L/h and at 450 °C. The representative chromatograms of HSG4112(S) and HSG4112(R) in rat and dog plasma are provided in Supplementary data (Figure S1). The validation for the quantitation method was conducted and the resulting data were satisfactory (Supplementary data). For metabolite profiling, an ACQUITY UPLC BEH C18 column (2.1 × 150 mm, 1.7 µm; Waters, Milford, MA, USA) was used and a gradient elution program was used as follows: 30%B to 75% B at 10 min, to 30% B at 10.1 min, and held at 30% B for 3 min. The metabolite levels were measured using selected ion monitoring based on the *m/z* values of the deprotonated ions of metabolites. Other mass spectrometer conditions were the same as above.

### 2.7. LC-QTOF/MS for Metabolite Analysis

The high performance liquid chromatography quadruple time of the flight mass spectrometer (LC-QTOF/MS) system consisted of an Agilent 1260 series binary pump HPLC system and an Agilent 6530 Q-TOF/MS/MS equipped with an electrospray ionization source (Agilent Technologies, Palo Alto, CA, USA). The column used for the separation was a Thermo Hypersil Gold column (2.1 × 150 mm, 3 μm; Thermo Fisher Scientific Inc., Waltham, MA, USA). Column temperature was maintained at 40 °C using a thermostatically controlled column oven. The HPLC mobile phases consisted of 0.1% formic acid in distilled water (A) and 90% acetonitrile in 0.1% formic acid (B). A gradient program was used for the HPLC separation with a flow rate of 0.2 mL/min. The initial composition of the mobile phase was 30% B and it was changed to 90% B over 13 min and followed by a 7 min re-equilibration to the initial condition. The entire column eluent was directly introduced into the mass spectrometer. Nitrogen was used both as the nebulizing gas at 20 psi and as the drying gas at a flow rate of 10 L/min at 300 °C. The mass spectrometer was operated in the negative ion mode in *m*/*z* 50–400.

### 2.8. Pharmacokinetic and Data Analysis 

All data were expressed as mean ± SD. Pharmacokinetic parameters were calculated by non-compartmental analysis using Pheonix WinNonlin (Ver. 6.2, Pharsight-A Certara Company, USA). The area under the concentration–time curve from time zero to the last measurable concentration (AUC_last_), area under the plasma concentration–time curve to the infinite time (AUC_inf_), maximum plasma concentration (*C*_max_), time to reach *C*_max_ (*T*_max_), terminal elimination half-life (*t*_1/2_), total body clearance (*C*_l_), apparent volume of distribution (*V*_z_), apparent volume of distribution at steady state (*V*_ss_), and equation for the mean residence time (MRT) were estimated by non-compartmental analysis of the plasma concentration versus time. The significance of the pharmacokinetic parameters was assessed using the paired Student’s *t*-test. The analysis was performed on log-transformed data. When *p* value was less than 0.05, it was judged to be significant.

## 3. Results

### 3.1. Analysis of HSG4112(S) and HSG4112(R) in Rat Plasma

**Figure 2:**
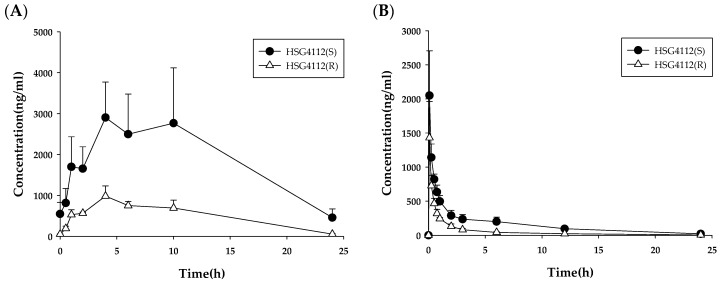
Plot for time-plasma concentration of HSG4112 after (**A**) oral (100 mg/kg/day, 28^th^ day, *n* = 3) and (**B**) iv (10 mg/kg, single dose, *n* = 3) administration in rats.

The time–plasma concentration plots of HSG4112(S) and HSG4112(R) are presented in Figure 2A, and the pharmacokinetic parameters are described in Table 1. The plasma concentration levels of HSG4112(S) were significantly higher than those of HSG4112(R) throughout all time points. The *C*_max_ of HSG4112(S) and HSG4112(R) were 2904.9 ng∙h/mL and 984.6 ng/mL, respectively. The area under the curve (AUC) of HSG4112(S) and HSG4112(R) were 45,733.3 and 12,190.6 ng∙h/mL, respectively. The pharmacokinetics of HSG4112(S) and HSG4112(R) after IV administration of HSG4112 were also investigated. The plasma concentration profiles of HSG4112(S) and HSG4112(R) after IV injection of HSG4112 at a dose of 10 mg/kg are presented in Figure 2B, and the pharmacokinetic parameters are described in Table 2. The stereoselective differences in plasma concentration were also observed in the IV administration. The AUC of HSG4112(S) and HSG4112(R) were 3806.7 ± 894.6 ng∙h/mL and 1390.4 ± 92.7 ng∙h/mL, respectively. Validation Data are presented in Tables S2–S5.

### 3.2. Analysis of HSG4112(S) and HSG4112(R) in Dog Plasma

**Figure 3:**
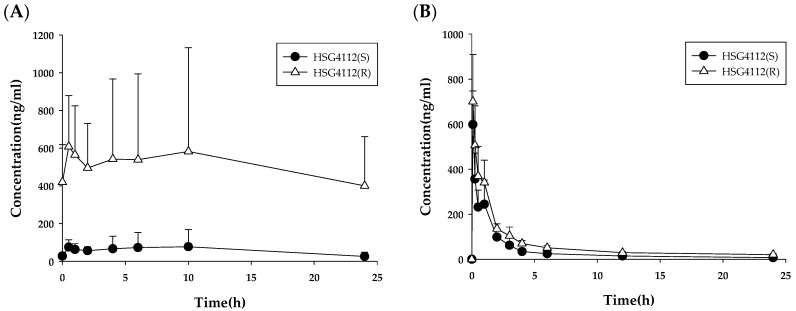
Plot for time–plasma concentration of HSG4112 after (**A**) oral (100 mg/kg/day, 28^th^ day, *n* = 3) and (**B**) iv (2 mg/kg, single dose, *n* = 2) administration in dogs.

The time–plasma concentration plots of HSG4112(S) and HSG4112(R) are presented in Figure 3A, and the pharmacokinetic parameters are described in Table 3. The *C*_max_ of HSG4112(S) and HSG4112(R) were 102.6 ± 69.6 and 707.6 ± 442.7, respectively. The AUC of HSG4112(S) and HSG4112(R) were 1408.4 ± 1418.0 and 12,324.8 ± 9715.7, respectively. The plasma concentration profiles of HSG4112(S) and HSG4112(R) after IV injection of HSG4112 at a dose of 2 mg/kg are presented in Figure 3B, and the pharmacokinetic parameters are described in Table 4. After IV injection of 2 mg/kg to beagle dogs, the AUC of HSG4112(S) and HSG4112(R) were 1002.6 ± 163.6 and 1837.4 ± 20.4 ng·h/mL, respectively. Validation Data are presented in Tables S6–S9.

### 3.3. Metabolic Stability

**Figure 4:**
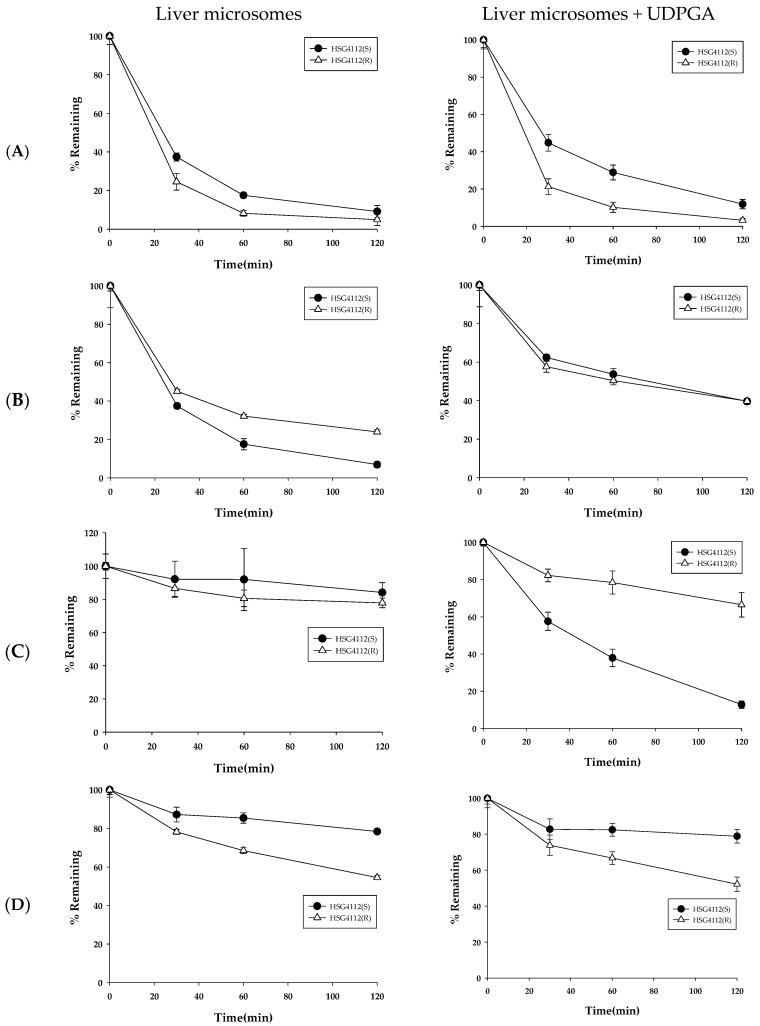
Metabolic stability of HSG4112 in (**A**) rat, (**B**) mouse, (**C**) dog, and (**D**) human liver microsomes (*n* = 3). UDPGA, uridine 5′-diphosphoglucuronic acid.

The results for the metabolic stability of HSG4112(S) and HSG4112(R) in rat (RLM), mouse (MLM), dog (DLM), and human liver microsomes (HLM) is shown in Figure 4 (left). The remaining amounts of HSG4112(S) at 120 min were 9.1 ± 3.2%, 6.9 ± 1.5%, 84.1 ± 6%, and 78.4 ± 0.5% in the RLM, MLM, DLM, and HLM, respectively. The half-life of HSG4112(S) was 15.5, 14.2, 664.8, and 424.6 min in the RLM, MLM, DLM, and HLM, respectively. The remaining amounts of HSG4112(R) at 120 min were 4.9 ± 3%, 23.9 ± 1.1%, 77.9 ± 2.9%, and 54.5 ± 1.1% in the RLM, MLM, DLM, and HLM, respectively. The half-life of HSG4112(R) was 8.2, 28.0, 376.8, and 133.9 min in the RLM, MLM, DLM, and HLM, respectively.

To investigate the effects of the phase II metabolism by glucuronidation, metabolic stability was tested after adding the glucuronidation cofactor, UDPGA. The results for the metabolic stability of HSG4112(S) and HSG4112(R) in rat, mouse, dog, and human liver microsomes with UDPGA are shown in Figure 4 (right). The remaining amounts of HSG4112(S) at 120 min were 11.9 ± 2.5%, 39.6 ± 1.5%, 12.8 ± 2%, and 78.9 ± 3.8% in the RLM, MLM, DLM, and HLM, respectively. The half-life of HSG4112(S) was 25.6, 64.3, 40.0, and 419.6 min in the RLM, MLM, DLM, and HLM, respectively. The remaining amounts of HSG4112(R) at 120 min were 3.2 ± 1.1%, 39.7 ± 0.9%, 66.5 ± 6.6%, and 52.2 ± 4% in the RLM, MLM, DLM, and HLM, respectively. The half-life values of HSG4112(R) were 9.1, 52.4, 226.8, and 150.5 min in the RLM, MLM, DLM, and HLM, respectively.

**Figure 5:**
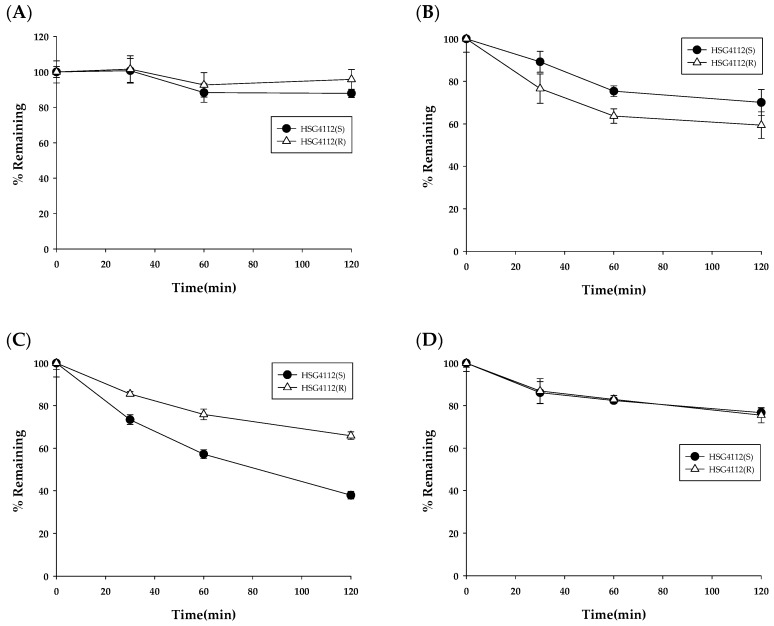
Metabolic stability of HSG4112 in (**A**) rat, (**B**) mouse, (**C**) dog, and (**D**) human intestinal microsomes (*n* = 3).

As glucuronidases are also predominantly distributed in the intestine, HSG4112 was tested in intestinal microsomes to confirm the metabolic stability results while considering glucuronidation. The results for the metabolic stability of HSG4112(S) and HSG4112(R) in rat (RIM), mouse (MIM), dog (DIM), and human intestinal microsomes (HIM) with UDPGA are shown in Figure 5. The remaining amounts of HSG4112(S) at 120 min were 87.9 ± 2.3%, 70.1 ± 6.1%, 37.9 ± 1.8%, and 76.7 ± 2.1% in RIM, MIM, DIM, and HIM, respectively. The half-life values of HSG4112(S) were 733.9, 247.9, 78.1, and 368.1 min in the RIM, MIM, DIM, and HIM, respectively. The remaining amounts of HSG4112(R) at 120 min were 95.8 ± 5.6%, 59.4 ± 6.3%, 65.9 ± 1.8%, and 75.5 ± 3.6% in RIM, MIM, DIM, and HIM, respectively. The half-life of HSG4112(R) was 1943.9, 136.7, 215.3, and 351.1 min in RIM, MIM, DIM, and HIM, respectively.

### 3.4. Metabolite Profile

**Figure 6:**
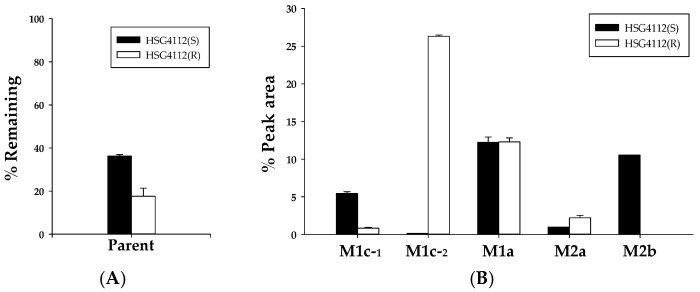
Metabolic profiles of HSG4112(S) and HSG4112(R) in rat liver microsomes. (**A**) Percent remaining amounts of HSG4112(S) and HSG4112(R). (**B**) Relative peak areas of the metabolites generated from HSG4112(S) and HSG4112(R). HSG4112(S) and HSG4112(R) were incubated separately in rat liver microsomes for 60 min and the resulting amount of the parent drug and its metabolites was measured based on peak area.

**Figure 7:**
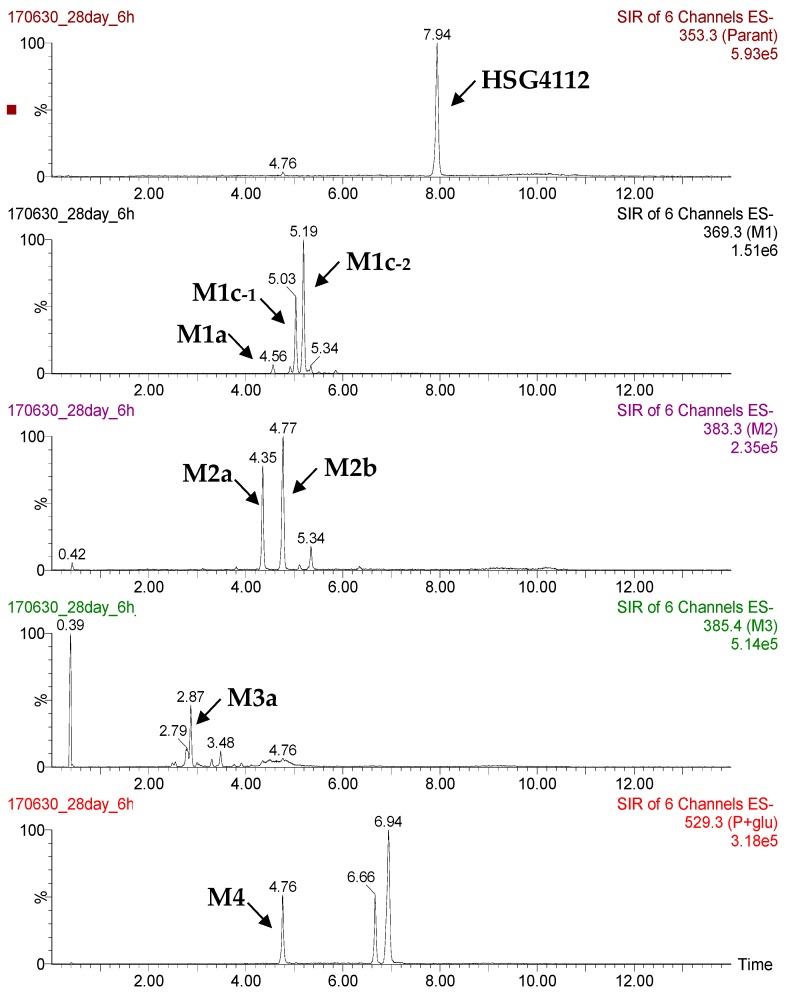
Representative extracted ion chromatograms of HSG4112 and its metabolites in rat plasma.

**Figure 8:**
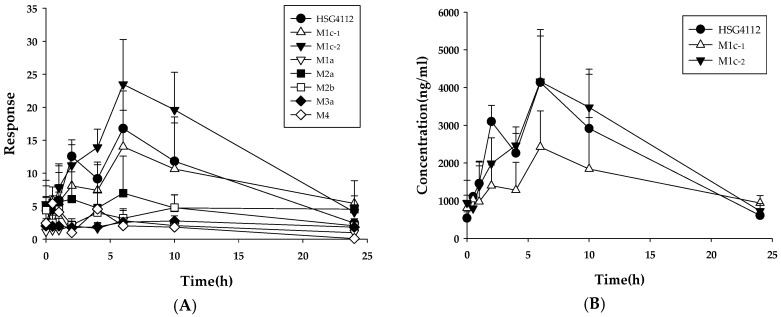

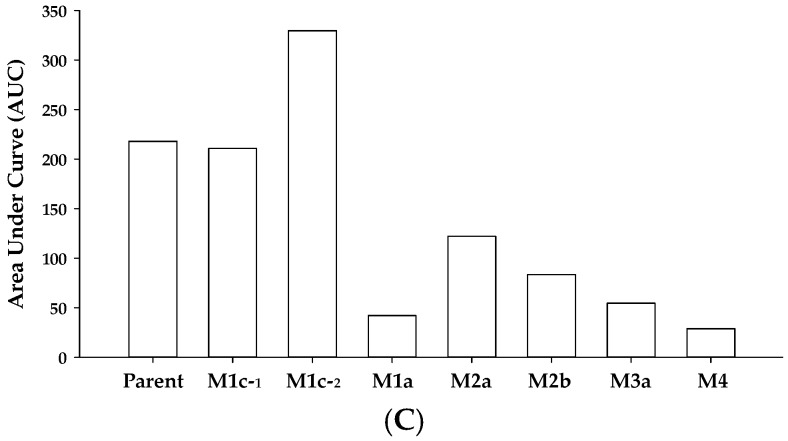
Metabolic profiles of HSG4112 in rat plasma after oral administration (100 mg/kg/day, 28th day, *n* = 3). (**A**) Plot for time-peak area of HSG4112 and its metabolites. (**B**) Plot for time-plasma concentration of HSG4112, M1c-1 and M1c-2. (**C**) Area under curve values of HSG4112 and its metabolites calculated from (**A**).

To elucidate the main factor responsible for the stereospecific pharmacokinetic properties in rats, the metabolite profiles of HSG4112(S) and HSG4112(R) were investigated. When HSG4112(S) and HSG4112(R) were incubated in the rat liver microsomes for 60 min, the remaining amounts of HSG4112(S) and HSG4112(R) were 36.2% and 17.6%, respectively (Figure 6A). HSG4112(S) and HSG4112(R) were metabolized to yield metabolites by oxidation (M1a ~ M2b). M1c-1 and M1c-2 were detected as one peak on a general C18 column but they were separated on a chiral column. Thus, M1c-1 and M1c-2 were supposed to be stereoisomers. The types of metabolites produced from the two isomers were similar, but the amounts produced were different. The predominant metabolites of HSG4112(S) were M1a and M2b, whereas those of HSG4112(R) were M1c-2 and M1a (Figure 6B). Subsequently, the metabolite profile was investigated in the rat plasma samples (on the 28^th^ day of an oral dose of 100 mg/kg/day). A total of seven metabolites were detected (Figure 7); a metabolite with hydroxyl and carbonyl groups (M3a) and glucuronide conjugate (M4) were additionally detected besides the metabolites observed in liver microsomes. Their peak area values were plotted according to the time (Figure 8A), and the concentration of the parent (as racemate), M1c-1, and M1c-2 were quantitated (Figure 8B). M1c-2 was shown to be the predominant metabolite (Figure 8C). The accurate mass data for each postulated metabolite is tabulated in Table 5. The tentative structures of HSG4112 metabolites are provided as Supplementary data (Figure S2).

**Figure 9:**
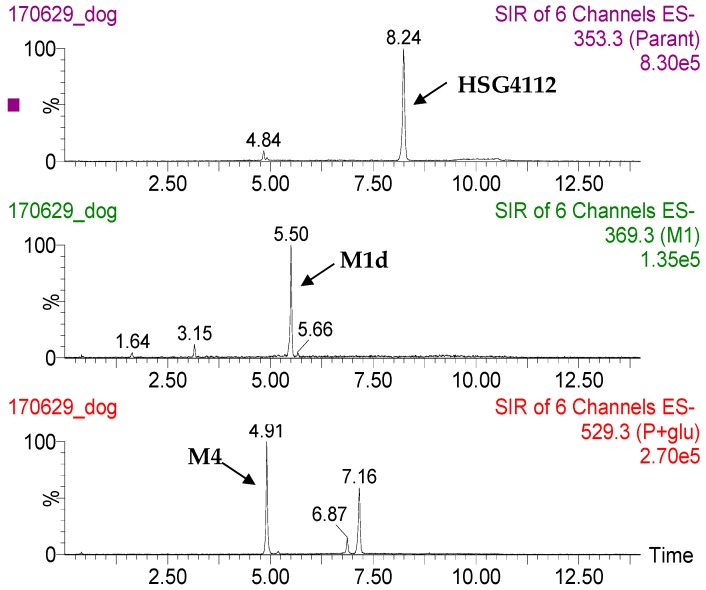
Representative extracted ion chromatograms of HSG4112 and its metabolites in dog plasma.

Meanwhile, the dog plasma sample showed only two metabolites (M1d and M4); M4, the glucuronide metabolite, was the predominant metabolite (Figure 9). Due to the unavailability of the sample and reference standards, the time–plasma concentration profile could not be obtained.

## 4. Discussion

In this study, concentration profiles of HSG4112(S) and HSG4112(R) after intravenous and/or oral administration of HSG4112 were investigated in rats and dogs, and the stereoselectivity in the metabolism of HSG4112 was investigated in vitro and in vivo. The resulting data showed a characteristic stereoselective pharmacokinetic pattern depending on the species.

When HSG4112(S) and HSG4112(R) were quantified in rat plasma, the concentration of HSG4112(S) generally measured higher, and, consequently, the AUC of HSG4112(S) was 4.9–7.8 times higher than that of HSG4112(R). This trend was also observed in the plasma samples from rats administered intravenously; the concentration ratio (S/R) over time was 1.4–4.8, and the AUC of HSG4112(S) was 2.7 times higher than that of HSG4112(R). Therefore, the systemic exposure of HSG4112(S) was significantly higher than that of HSG4112(R) in rats, indicating stereospecificity in the pharmacokinetics. This stereoselective pharmacokinetic property in rats is supposedly due to the stereoselective metabolism of HSG4112. The possible metabolic pathways of HSG4112 isomers in rats are presented in Figure S3. The metabolic stability data and the metabolite profiles in rat plasma showed that HSG4112(R) is metabolized more extensively than HSG4112(S), and their main metabolic pathways are different. The metabolic profile data suggested that the formation of M1c is predominantly responsible for the stereoselective pharmacokinetics of HSG4112.

When HSG4112 was orally administered to beagle dogs, the concentration ratio (S/R) over time was 0.06–0.24, and the concentration of HSG4112(R) measured at a much higher level. The AUC ratio of HSG4112(S) and HSG4112(R) was 0.12–0.13 in the oral administration group. In addition, when HSG4112 was intravenously administered to beagle dogs, the concentration ratio (S/R) over time was 0.3–0.9, and the AUC ratio of HSG4112(S) and HSG4112(R) was 0.5. Therefore, the systemic exposure of HSG4112(R) was significantly higher than HSG4112(S) in beagle dogs, indicating stereospecificity in the pharmacokinetics. Interestingly, the predominant isomer form in dogs was opposite to that of rats. The metabolic stability data for phase I metabolism did not exhibit any difference between HSG4112(S) and HSG4112(R). However, the metabolic stability data including glucuronide formation showed significant differences between HSG4112(S) and HSG4112(R); the metabolic rate of HSG4112(S) was much higher than that of HSG4112(R). This metabolism pattern was also obviously observed in dog intestinal microsomes, which also have high glucuronidase activities. This revealed that the stereoselective glucuronide formation is mainly responsible for the stereoselective pharmacokinetics of HSG4112 in dogs. This was also supported by the in vivo metabolism profile data of dog plasma; the glucuronide metabolite (M4) was found to be the predominant metabolite in dog plasma. Notably, the longer terminal half-life was observed after oral administration compared with IV injection in dogs. Thus, it is supposed that HSG4112 has a general-case pharmacokinetic property in rats but a flip-flop pharmacokinetic property in dogs. This phenomenon occurs when the absorption rate of drugs is much slower than the elimination rate [11,12]. In this case, bioavailability factors such as the absorption rate and extent mainly affect the terminal slope of oral administration than clearance and volume of distribution.

Glabridin, the motive compound of HSG4112, has the R configuration at carbon C-3. According to the previous report, the systemic bioavailability of glabridin was very low (about 7.5% in rats). Glabridin is mainly metabolized by glucuronidases in the intestine and liver, and the first-pass effects of glucuronidation are one of the main factors responsible for the low oral bioavailability of glabridin. Guo et al. investigated the tissue and species differences in the glucuronidation of glabridin. In their study, glabridin was metabolized to yield two glucuronide metabolites: M1 and M2. M2 formation was predominant whereas the formation of M1 was negligible in most species tested. The authors could not determine the exact position of glucuronidation for M1 and M2, but they suggested that M2 might have a glucuronide moiety at the C-4 hydroxyl group in the B ring based on the earlier reports. The C-4 hydroxyl group of HSG4112 is masked by an alkyl chain. This is the reason why the glucuronidation was not the main metabolic pathway in humans and rats in our study with HSG4112. It is notable that dog liver microsomes showed the highest value of the intrinsic clearance by M1 formation (glucuronidation at the C-2 hydroxyl group) in glabridin. This result agrees with our finding that the formation of the glucuronide metabolite predominantly occurred in dog liver and intestinal microsomes. The results by Guo et al. showed that the metabolic clearance by glucuronidation both for M1 and M2 was negligible in humans. This is also consistent with our data that the contribution of glucuronidation to the metabolic clearance of HSG4112 was minimal.

Stereoselectivity for HSG 4112 metabolism was also shown in human liver microsomes. The metabolism pattern in human liver microsomes is somewhat close to that in rat liver microsomes. Thus, this is supposedly due to CYP-mediated metabolism rather than glucuronidation. Although the metabolic rate in humans is lower than that in other species, the present data suggests that stereoselective pharmacokinetics by stereoselective metabolism (i.e., higher exposure of the (S)-isomer) could be shown in the clinical trial of HSG4112. Another factor that should be considered regarding stereoselective pharmacokinetics is the possibility of interconversion between the isomers. To address this issue, HSG4112 isomers were analyzed after oral administration of each isomer to rats. When one isomer was administered, the other isomer was not detected (data not shown). This suggests that interconversion between isomers does not occur at least in rats. Further investigation should be followed in humans.

In conclusion, the present study demonstrates that HSG4112 showed species-specific stereoselective pharmacokinetics. Notably, the pharmacokinetic stereoselectivity of HSG4112 isomers showed opposing patterns between rats and dogs. This is because the major metabolic pathways involved in the clearance of HSG4112 are different in rats and dogs. Each metabolic enzyme may have different stereoselectivity. We presented the possible mechanisms for these stereoselective pharmacokinetic patterns by the metabolite profiling data in vitro and in vivo. These results will provide helpful information for understanding the pharmacological and toxicological effects of HSG4112, depending on its configurations. In addition, caution should be taken in extrapolating preclinical data with different experimental animal models to clinical data for humans.

## Figures and Tables

**Table 1:** Pharmacokinetic parameters of HSG4112(S) and HSG4112(R) after PO administration at a repeated dose of 100 mg/kg/day to rats.

PK Parameter ^a^	Rat 100 mg/kg (PO, 28th Day)	
	HSG4112(S)	HSG4112(R)
*T*_max_ (h)	4	4
*C*_max_ (ng/mL)	2904.9	984.6
AUC_last_ (ng·h/mL)	45,733.3	12,190.6
*T*_1/2_ (h)	7.3	4.7
AUC_inf_ (ng·h/mL)	50,555.8	12,558.1
MRT (h)	8.8	7.8

^a^ The blood samples were collected by a sparse sampling method from six rats (Table S1). Accordingly, the pharmacokinetic parameters were calculated from the mean plasma concentration data.

**Table 2:** Pharmacokinetic parameters of HSG4112(S) and HSG4112(R) after IV administration at a single dose of 10 mg/kg to rats.

PK Parameter	Rat 10 mg/kg (IV, *n* = 3)	
	HSG4112(S)	HSG4112(R)
*C*_max_ (ng/mL)	2049.7 ± 654.0	1431.7 ± 530.5 ***
AUC_last_ (ng·h/mL)	3806.7 ± 894.6	1390.4 ± 92.7 **
*T*_1/2_(h)	5.3 ± 1.0	5.9 ± 0.4
AUC_inf_ (ng·h/mL)	3976.5 ± 1027.7	1436 ± 98.7 **
*V*_z_ (mL/kg)	9667.6 ± 697.8	29,483.8 ± 393.6 ***
*C*_l_ (mL/h/kg)	1318.8 ± 360.1	3493.4 ± 247.2 **
MRT (h)	5.4 ± 0.9	3.9 ± 0.4 *
*V*_ss_ (mL/kg)	16374 ± 1281	33,462 ± 4228 ***

*: *p* < 0.05, **: *p* < 0.01, ***: *p* < 0.005 versus HSG4112(S).

**Table 3:** Pharmacokinetic parameters of HSG4112(S) and HSG4112(R) after PO administration at a repeated dose of 100 mg/kg/day to dogs.

PK Parameter	Dog 100 mg/kg (PO, 28th day, *n* = 3)	
	HSG4112(S)	HSG4112(R)
*T*_max_ (h)	4.2 ± 5.1	3.7 ± 5.5
*C*_max_ (ng/mL)	102.6 ± 69.6	707.6 ± 442.7 ***
AUC_last_ (ng·h/mL)	1408.4 ± 1418.0	12324.8 ± 9715.7 ***
*T*_1/2_ (h)	17.5 ± 4.5	49.4 ± 19.1
AUC_inf_ (ng·h/mL)	NA	24329.9 ± 6226.4 **
MRT (h)	9.2 ± 0.4	11.1 ± 0.2 *

*: *p* < 0.05, **: *p* < 0.01, ***: *p* < 0.005 versus HSG4112(S). NA: not available.

**Table 4:** Pharmacokinetic parameters of HSG4112(S) and HSG4112(R) after IV administration at a single dose of 2 mg/kg to dogs.

PK Parameter	Dog 2 mg/kg (IV, *n* = 2)	
	HSG4112(S)	HSG4112(R)
*C*_max_ (ng/mL)	598.8 ± 149.2	700.9 ± 208.7
AUC_last_ (ng·h/mL)	902.7 ± 165.2	1519.0 ± 95.1
*T*_1/2_(h)	10.3 ± 1.5	12.0 ± 1.6
AUC_inf_ (ng·h/mL)	1002.6 ± 163.6	1837.4 ± 20.4
*V*_z_ (mL/kg)	15180.7 ± 4635.6	9437.1 ± 1333.6
*C*_l_ (mL/h/kg)	1008.6 ± 164.5	533.1 ± 5.2
MRT (h)	4.4 ± 0.3	5.7 ± 0.1
*V*_ss_ (mL/kg)	8047.5 ± 1885.4	6500.5 ± 1157.4

**Table 5:** Accurate mass data for HSG4112 and its postulated metabolites in rats.

Metabolites	ΔM	Theoretical Mass [M-H]^−^	Measured Mass [M-H]^−^	Error (ppm)
HSG4112	-	353.1758	353.1756	−0.57
M1a	+16	369.1707	369.1712	1.35
M1c-1	+16	369.1707	369.1718	2.98
M1c-2	+16	369.1707	369.1718	2.98
M2a	+30	383.1500	383.1499	−0.26
M2b	+30	383.1500	383.1531	8.09
M3a	+30	385.1657	385.1662	1.30
M4	+30	529.2079	529.2071	−1.51

## Supplementary Materials

The following are available online at https://www.mdpi.com/1999-4923/12/2/127/s1, Figure S1: Representative chromatograms of HSG4112(S) and HSG4112(R) in (A) rat (oral, 100 mg/kg, 6h) and (B) dog (oral, 100 mg/kg, 6h) plasma, Figure S2: Proposed chemical structures of HSG4112 metabolites, Figure S3: Postulated metabolic pathways of HSG4112 isomers, Table S1: Blood drain time points for oral pharmacokinetic analysis in rats (repeated dose, 100 mg/kg/day, 28th day), Table S2: Linearity of HSG4112(S) and HSG4112(R) in rat plasma (*n* = 3), Table S3: Intra-day and inter-day accuracy and coefficient of variation for determination of HSG4112(S) and HSG4112(R) in rat plasma, Table S4: Matrix effect, recovery and process efficiency data for HSG4112(S) and HSG4112(R) in rat plasma (*n* = 3), Table S5: Stability of HSG4112(S) and HSG4112(R) in rat plasma, Table S6: Linearity of HSG4112(S) and HSG4112(R) in dog plasma, Table S7: Intra-day and inter-day accuracy and coefficient of variation for determination of HSG4112(S) and HSG4112(R) in dog plasma, Table S8: Matrix effect, recovery and process efficiency data for HSG4112(S) and HSG4112(R) in dog plasma (*n* = 3), Table S9: Stability of HSG4112(S) and HSG4112(R) in dog plasma.
