# Supplementary material for: Species Differences in Stereoselective Pharmacokinetics of HSG4112, A New Anti-Obesity Agent
Source: Pharmaceutics. 2020 Feb 3;12(2):127. doi: 10.3390/pharmaceutics12020127 (PMC7076457; doi:10.3390/pharmaceutics12020127)
Supplement: Supplementary file 1 [file pharmaceutics-12-00127-s001.pdf]

pharmaceutics

Supplementary Materials: Species Differences in Stereoselective Pharmacokinetics of HSG4112, A New Anti-Obesity Agent

In Yong Bae, Min Sun Choi, Young Seok Ji, Sang-Ku Yoo, Kyungil Kim, Hye Hyun Yoo

1. Plasma Sample Information

(1) Rat nonclinical formulation repeated dosing plasma samples (Sparse sampling)
Animals: Sprague-Dawley male rats (6-week old, $n = 6$)
Oral administration (100 mg/kg) (No. 1601 ~ 1606) (28 day)
Blood sampling points: 0. 0.5, 1, 2, 4, 6, 10, 24 h (Table S1)

Table S1. Blood drain time points for oral pharmacokinetic analysis in rats (repeated dose, 100 mg/kg/day, 28th day).

Time (h)	Rat #1601	Rat #1602	Rat #1603	Rat #1604	Rat #1605	Rat #1606
0	○	○	○			
0.5				○	○	○
1	○	○	○			
2				○	○	○
4	○	○	○			
6				○	○	○
10	○	○	○			
24				○	○	○

(2) Rat nonclinical formulation single dosing plasma samples ($n = 3$)
Animals: Sprague-Dawley male rats (6-week old)
Intravenous injection (10 mg/kg) (No. 1403~1405)
Blood sampling points: 0, 0.083, 0.25, 0.5, 0.75, 1, 2, 3, 6, 12, 24 h

(3) Dog nonclinical formulation repeated dosing plasma samples ($n = 3$)
Animals: Male beagle dogs (6-month old)
Oral administration (100 mg/kg) (No. 1201~1203) (28 day)
Blood sampling points: 0, 1, 2, 4, 6, 10, 12, 24 h

(4) Dog nonclinical formulation single dosing plasma samples ($n = 2$)
Animals: Male beagle dogs (6-month old)
Intravenous injection (2 mg/kg) (No. 1101, 1102)
Blood sampling points: 0, 0.083, 0.25, 0.5, 1, 2, 3, 4, 6, 12, 24 h

2. Analytical Method Validation Data

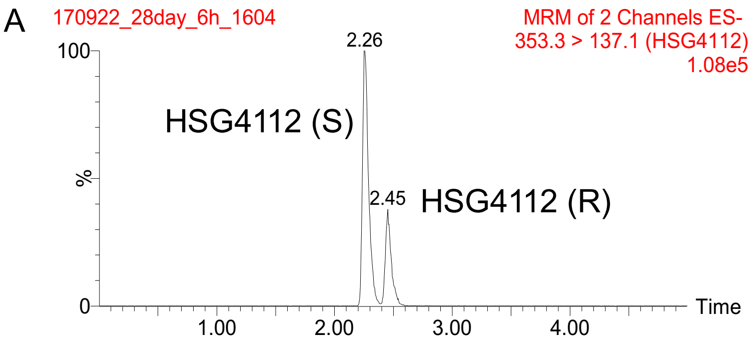

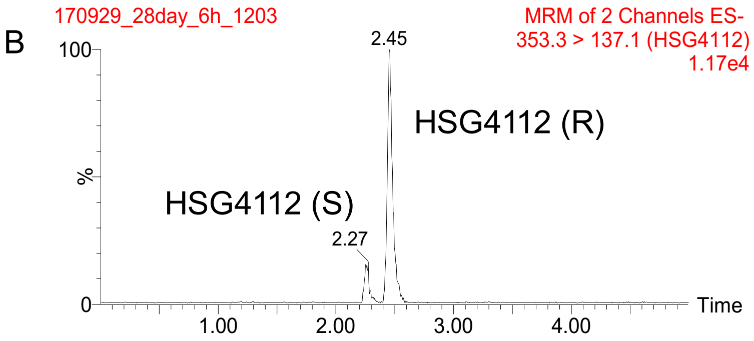

Figure S1. Representative chromatograms of HSG4112(S) and HSG4112(R) in (A) rat (oral, 100 mg/kg, 6h) and (B) dog (oral, 100 mg/kg, 6h) plasma.

Table S2. Linearity of HSG4112(S) and HSG4112(R) in rat plasma (n = 3).

Specified Conc.(ng/mL)	HSG4112(S)			HSG4112(R)		
	Calculated Conc.(ng/mL)	Accuracy (%)	CV (%)	Calculated Conc.(ng/mL)	Accuracy (%)	CV (%)
5	5	99.3	1.2	4.8	96	15
20	18.8	94	14.9	20.2	100.8	11.8
50	47.9	95.9	6.3	51.5	103.1	12.2
100	97.2	97.2	6.5	104.4	104.4	5.2
500	502.7	100.5	3.5	505.5	101.1	5.1
1000	969.8	97	3.2	919.6	92	10.8
2000	2104.7	105.2	6.9	2020.8	101	1.4
5000	5083.5	101.7	2.2	4983.7	99.7	0.5

Table S3. Intra-day and inter-day accuracy and coefficient of variation for determination of HSG4112(S) and HSG4112(R) in rat plasma.

Compound	QC Level	Conc. (ng/mL)	Intra run (n = 5)		Inter run (n = 5)	
			Accuracy (%)	CV (%)	Accuracy (%)	CV (%)
HSG4112(S)	LOQ	5	102.4	4.7	103.7	3.4
	LOW	15	100.1	9.3	103.4	4.5
	MID	400	104.1	6.4	102.4	2.8
	HIGH	4000	107.4	3.1	101.4	5.2
HSG4112(R)	LOQ	5	97.6	17.2	99.2	7.1
	LOW	15	115.3	4.9	102.1	5.7
	MID	400	98.2	6.4	101.9	4.4
	HIGH	4000	110	2.6	102.1	7.1

Table S4. Matrix effect, recovery and process efficiency data for HSG4112(S) and HSG4112(R) in rat plasma (n = 3).

Compound	Theoretyical Conc. (ng/mL)	Matrix Effect (%)	Recovery (%)	Process Efficiency (%)
HSG4112(S)	15	89.8 ± 7.2	101.9 ± 7.4	91.2 ± 5.7
	4000	95.6 ± 3	94.8 ± 5.1	90.7 ± 5.5
HSG4112(R)	15	111.7 ± 5.9	74.1 ± 7.8	82.6 ± 8.6
	4000	118.5 ± 1.9	93.4 ± 2.3	110.6 ± 1.5

Table S5. Stability of HSG4112(S) and HSG4112(R) in rat plasma.

Stability	Conc. (ng/mL)	% of Control (n = 3)			
		HSG4112(S)		HSG4112(R)	
		Accuracy	CV	Accuracy	CV
Short term stability	15	100	4.8	100.2	1.4
	4000	89.6	6.4	88.5	7.2
Long term stability	15	94.9	5.7	98.0	10.9
	4000	110.0	1.0	112.5	0.3
Freeze-thaw stability	15	107.3	1.1	115.3	15.1
	4000	107.6	3.6	101	2.9
Processing stability	15	112	2.5	103.3	6.1
	4000	95.8	3.8	94.1	3.2

Table S6. Linearity of HSG4112(S) and HSG4112(R) in dog plasma.

Specified Conc.(ng/mL)	HSG4112(S)			HSG4112(R)		
	Calculated Conc.(ng/mL)	Accuracy (%)	CV (%)	Calculated Conc.(ng/mL)	Accuracy (%)	CV (%)
5	5.1	102.7	3	4.3	85.3	8.9
20	19.1	95.5	2.3	20.3	101.7	17
50	48.7	97.4	6.9	52.4	104.7	8.1
100	102.2	102.2	3.8	102.5	102.5	1.7
500	501.5	100.3	2.4	518	103.6	2.6
1000	1028.3	102.8	4.4	1029.7	103	5.9
2000	2045.5	102.3	3.4	2017.4	100.9	2.6
5000	4813.2	96.3	2.1	4930.8	98.6	0.7

Table S7. Intra-day and inter-day accuracy and coefficient of variation for determination of HSG4112(S) and HSG4112(R) in dog plasma.

Compound	QC Level	Conc.(ng/mL)	Intra Run (n = 5)		Inter Run (n = 5)	
			Accuracy (%)	CV (%)	Accuracy (%)	CV (%)
HSG4112(S)	LOQ	5	96.8	5.6	99.6	6.1
	LOW	15	99.6	4	105.5	4.7
	MID	1500	102.5	3.2	110.1	4
	HIGH	4000	101.7	1.3	101.2	1.6
HSG4112(R)	LOQ	5	107.2	12.7	93.7	7.2
	LOW	15	106.4	3.8	102.7	4.7
	MID	1500	101.5	2.6	107.5	5.5
	HIGH	4000	101	1.3	100.9	1.6

Table S8. Matrix effect, recovery and process efficiency data for HSG4112(S) and HSG4112(R) in dog plasma (n = 3).

Compound	Theoretyical Conc. (ng/mL)	Matrix Effect (%)	Recovery (%)	Process Efficiency (%)
HSG4112(S)	15	104.3±10.5	91.9±9.9	95.1±5.4
	4000	118.4±1.9	87.9±1.6	104±2.5
HSG4112(R)	15	112.4±7.9	92.1±5	103.4±7.6
	4000	116±3.2	90±1.5	104.4±2.9

Table S9. Stability of HSG4112(S) and HSG4112(R) in dog plasma.

Stability	Conc. (ng/mL)	% of Control (n=3)			
		HSG4112(S)		HSG4112(R)	
		Accuracy	CV	Accuracy	CV
Short term stability	15	105.3	9.4	100.4	3.3
	4000	90.8	2.7	89.5	0.4
Long term stability	15	99.8	7.4	98.0	5.2
	4000	110.6	0.4	113.2	2.0
Freeze-thaw stability	15	100.4	8.8	101.8	13.4
	4000	112.7	2.1	105.3	0.8
Processing stability	15	104.4	10.3	106.2	7.5
	4000	92.7	1.8	92.4	0.7

3. Metabolism of HSG4112

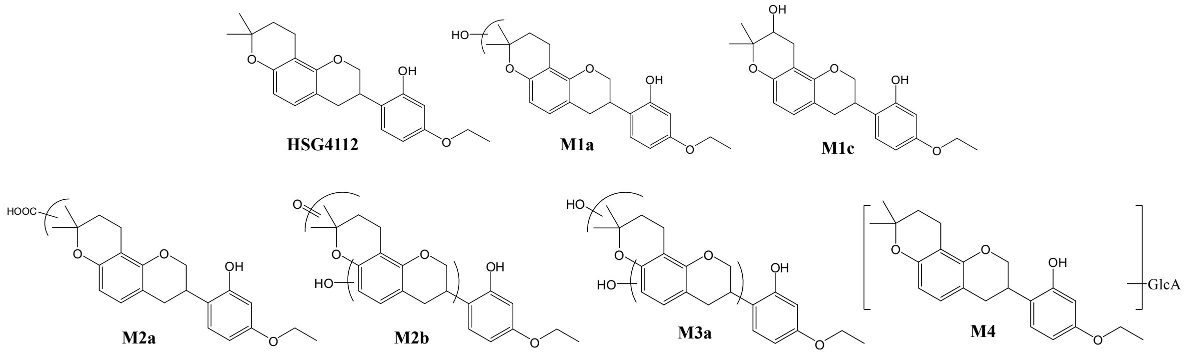

Figure S2. Proposed chemical structures of HSG4112 metabolites.

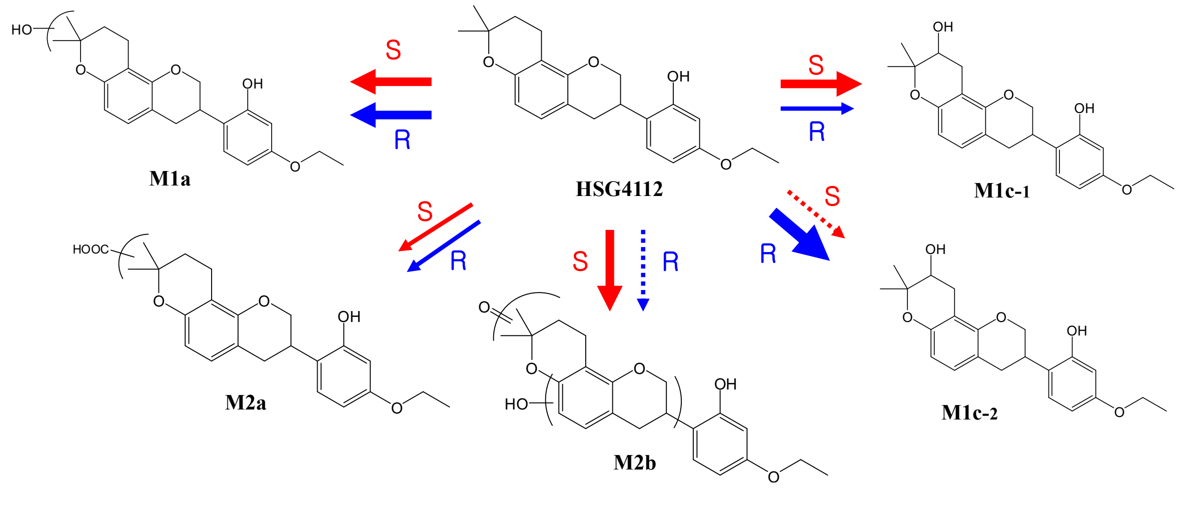

Figure S3. Postulated metabolic pathways of HSG4112 isomers.
